# Evaluating predictive factors for toxicities experienced by head & neck cancer patients undergoing radiotherapy

**DOI:** 10.1186/s12967-021-03047-2

**Published:** 2021-09-07

**Authors:** Xenia Ray, Whitney Sumner, Leisa Sutton, Parag Sanghvi, Ida Deichaite, Vitali Moiseenko

**Affiliations:** 1 Department of Radiation Medicine and Applied Sciences, University of California San Diego, La Jolla, CA USA; 2 Moores Cancer Center, University of California San Diego, La Jolla, CA USA

**Keywords:** Predictive biomarkers of radiation toxicity, Head and neck squamous cell carcinoma, RT dosimetric data, Human papilloma virus

## Abstract

**Purpose:**

The purpose of this study was to evaluate if HPV status serves as an independent predictor of early and late dysphagia outcomes when considered alongside standard patient characteristics and dose metrics for head and neck cancer patients treated with radiotherapy.

**Methods and materials:**

The age, sex, smoking history, cancer type (oropharyngeal vs non-oropharyngeal), HPV status, and early and late dysphagia outcomes were obtained for 99 retrospective head and neck cancer patients treated at our clinic with radiotherapy. Additionally for each patient, the mean radiation dose to the pharynx, superior/middle/inferior pharyngeal constrictor muscles, and cricopharyngeus was calculated. The predictive power of these clinical characteristics and radiation metrics was evaluated using chi-square tests for categorical variables and *t*-tests for continuous variables. Then multi-variate logistic models were built for each outcome using a single dose metric at a time, and either HPV status, cancer type, or both. Multi-variate models were built using both top-down and bottom-up technique to establish the most predictive independent covariates.

**Results:**

In the univariate analysis for early dysphagia, cancer type (p = 0.04) and four dose metrics (p ≤ 0.02) were significantly associated with outcome, while for late dysphagia, only cancer type (p = 0.04) was associated with outcome. In the multivariate analysis for early dysphagia, cancer type, smoking history, and mean dose to the five structures were consistently selected as covariates. For late dysphagia, either HPV status or cancer type was selected in each model and the mean dose to the cricopharyngeus was selected in one model.

**Conclusion:**

While HPV is a known contributing factor for tumor prognosis in oropharyngeal cancers, its role in normal tissue toxicities for head and neck cancers has not previously been evaluated. Our results indicate having an oropharyngeal cancer may increase a patient’s risk of high-grade early and late dysphagia while HPV status was seldom selected.

## Background

Radiotherapy (RT) is used as part of a curative treatment regimen for 75% of head and neck squamous cell carcinoma (HNSCC) patients [1]. The last few decades have seen marked improvements in outcomes in patients treated with RT for advanced HNSCC. Specifically, locoregional control at five years increased from approximately 30% in the 1980s to approximately 80% at the present time [2]. Several factors are responsible for these improvements including advancements in diagnostic imaging with the addition of positron emission tomography as well as innovations in radiation treatment delivery with the advent of intensity modulated radiation therapy (IMRT) and image-guided radiation therapy (IGRT) [3–5]. Additionally, optimized combinatorial strategies of chemoradiotherapy have further shaped the standard of care for HNSCC patients [6]. The next steps in optimization for the management of HNSCC have become increasingly reliant on biologic factors including tumor hypoxia and association with human papilloma virus (HPV) [7]. It is well-established that HPV-related oropharyngeal squamous cell carcinoma (OPSCC) represents a distinct entity in terms of both tumor and normal tissue response [8, 9]. The next generation of radiotherapeutic strategies in HNSCC now hinges on identifying additional tumor and patient-specific characteristics that are predictive for response in both tumors and surrounding critical structures [2].

Personalized RT has been a long-standing and elusive goal in the oncology community. It is appreciated that current prescribed doses and planning objectives to spare normal tissues are population-based. As a result, patients exhibit variable responses in terms of both tumor control and normal tissue toxicities that are, as yet challenging to predict. Is it therefore critical to establish biomarkers and/or patient-specific parameters that can accurately predict both tumor response and normal tissue outcomes in order to guide patient management. This is particularly critical as we now have the technology to facilitate daily adaptation of radiation therapy plans (e.g., Varian Ethos™ Therapy). Establishing clear criteria for patients at high risk for RT-related toxicity could allow us to optimize patient selection for this new, resource-intensive technology by clearly defining the patients who are likely to receive the greatest benefit from daily adaptation.

Thus far, this optimization has focused on establishing predictors of individual response has focused on tumor response. While certain tumor-specific features, e.g., hypoxia, can be assessed using modern imaging, accounting for genomic signatures is challenging. It has been demonstrated that overall dose–response for tumor control is a superposition of dose–responses which can be obtained for patient groups stratified according to tumor cell radiosensitivity [10]. On the other hand, normal tissue response has been demonstrated to be mediated by biological factors at cellular and molecular levels [11] including an argument that toxicity from radiation is genetically predetermined [12]. While the search for detailed genetic signatures for tumor and normal tissue response is ongoing, some patient-specific characteristics are readily available. In addition to general patient-specific data, (e.g., age, sex, smoking habits), HPV status is typically known. Accounting for HPV status has been tested in clinical trials evaluating the efficacy of replacing cisplatin with less toxic cetuximab [13] or reducing radiotherapy dose [14]. The impact of HPV status has been demonstrated for normal tissue toxicity, and HPV-positive patients were reported to exhibit higher rates of early mucositis [15]. However, accounting for HPV, or any other biomarker, has to be made with caution as it must hold independent predictive power.

The purpose of this study was to evaluate if HPV status serves as an independent predictor when considered in combination with standard patient characteristics for normal tissue dysphagia outcomes for head and neck cancer patients treated with radiotherapy. If found, these characteristics could be used to guide the management of patients in our clinic, particularly the stratification of adapted and non-adapted courses of radiation therapy.

## Methods

### Patient data

This retrospective analysis was approved by our institutional review board (UCSD HRPP#200495). To evaluate the association between clinical characteristics and normal tissue outcomes, we acquired patient characteristics and radiation data for 114 retrospective HNSCC patients treated at our institution between 2014 and 2019. Patient exclusion criteria included receiving more than one course of radiotherapy, treatment with non-standard dose fractionation (e.g., quadshot of 14 Gy in four fractions), and having an indistinguishable pharynx due to the extent of disease. Implementing these criteria left a total of 99 patients for analysis. From a review of each patient’s clinical chart, we obtained characteristics that may affect their outcomes: age, sex, smoking history, cancer type, and HPV status. To determine their normal tissue outcomes, quantitative scores were taken directly from chart review. Patient charts were utilized to report early and late RT adverse event (rtAE) endpoints for dysphagia. Toxicities were recorded using Common Terminology Criteria for Adverse Events (CTCAE) grades of 1–5, and were scored and reported by the treating physician on the day of service during RT and in routine follow-up as part of the standard clinical process at our institution. Early toxicity endpoints were recorded as the highest CTCAE grade experienced during RT or within 6 weeks of completing RT. Late toxicity endpoints were recorded as the highest CTCAE grade experienced from six months post-RT to the time of most recent follow up. The full list of characteristics for this cohort are displayed in Table 1.

**Table 1:** Patient characteristics

Characteristics	Patients (n = 99)
Age	
< = 65	49
> 65	50
Sex	
Male	78
Female	21
Smoking	
< 10 pack years	61
> = 10 pack years	38
HPV status	
Positive	55
Negative	44
Cancer type	
Oral cavity	19
Oropharynx	63
Larynx	8
Hypopharynx	5
Nasal cavity	1
Parotid gland	2
Trachea	1
Late dysphagia score	
0	22
1	45
2	16
3	16
4	0
5	0
Early dysphagia score	
0	13
1	31
2	32
3	22
4	1
5	0

To acquire radiation dose metrics for the analysis, a single trained medical professional evaluated the contours for relevant structures in each patient’s plan. When contours were missing or suboptimal, this same professional created a new structure that followed our contouring guidelines. Structures that were evaluated were the pharynx, superior pharyngeal muscles (PGM_sup), middle pharyngeal muscles (PGM_mid), inferior pharyngeal muscles (PGM_inf), and cricopharyngeus. The mean dose was then extracted for each of these structures from the treated plan. Mean dose was used for both the pharynx and sub-pharyngeal muscles as it has been linked to dysphagia in the QUANTEC reviews [16, 17]. Specifically, QUANTEC recommended that the pharynx mean dose should be kept < 50 Gy to keep incidence of symptomatic dysphagia < 20% and at our own clinic we use a planning goal of < 45 Gy for the pharynx minus PTV structure. Many patients had also received a mid-treatment replan. In those instances, the mean doses from the original plan and revised treatment plan were scaled by the number of treated fractions and summed.

### Statistical analysis

For the statistical analysis, we binarized early and late dysphagia from 0–2 versus 3–5. The relationship between categorical variables and each outcome was analyzed with a chi-square test where p-values < 0.05 were significant. Categorical variables were age (< 65 years or ≥ 65 years), sex (male or female), smoking history (< 10 pack years or ≥ 10 pack years), HPV status (positive or negative), and cancer type (oropharyngeal or non-oropharyngeal). Cancer type was converted to a binary variable differentiating only between oropharyngeal and non-oropharyngeal due to the small numbers of patients with certain non-oropharyngeal cancers (e.g., Tracheal with n = 1). The relationship between mean dose metrics and each outcome was evaluated using a *t*-test with p-values < 0.05 for significance.

Then multi-variate logistic models were built for each binarized outcome as a function of categorical variables (age, sex, smoking history), a single dose metric at a time, and either HPV status, cancer type, or both HPV status and cancer type. Dose metrics were handled separately because they were not independent of each other. HPV status and cancer type were evaluated separately and combined because HPV status is known to be heavily associated with oropharyngeal cancers. Each multi-variate logistic model was built using both a top-down and bottom-up modeling techniques to establish the most predictive independent covariates. Both the top-down and bottom-up methods were used to evaluate the consistency of covariate selection. Covariates with p-values < 0.05 were considered significant predictors in each model.

## Results

**Fig. 1:**
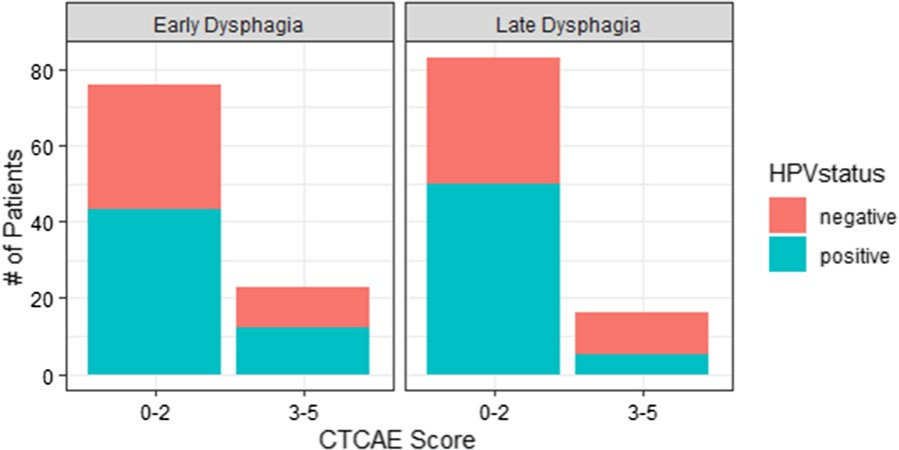
Incidence of HPV status in patients with low-grade (CTCAE 0–2) and high-grade (CTCAE 3–5) early and late dysphagia

Results of the chi-square tests are seen in Table 2. For early dysphagia only cancer type (oropharyngeal or non-oropharyngeal) was significantly associated with outcome though smoking history had a marginal p-value < 0.1. For late dysphagia, cancer type was also significantly associated with outcome and HPV status was the only other variable with a p-value < 0.1. Figure 1 shows the distribution of patients by outcome and HPV status.

**Table 2:** Chi-square test results for early and late dysphagia outcomes

Categorical covariates	Chi-square p-value (early dysphagia)	Chi-square p-value (late dysphagia)
Age (>/< 65 years)	0.96	0.75
Sex (M/F)	0.35	0.16
Smoking (>/< 10 PY)	0.07	0.84
HPV status (±)	0.89	0.06
Oropharyngeal cancer (Y/N)	0.04	0.04

**Fig. 2:**
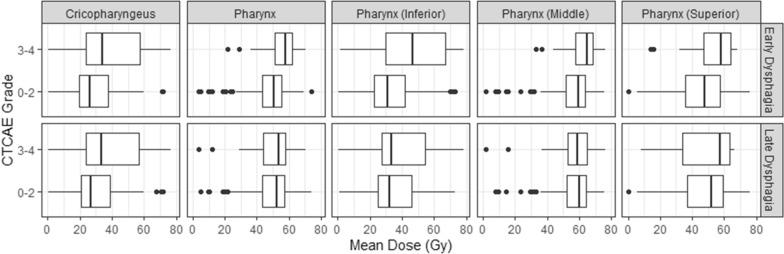
Distribution of mean dose values per structure for each outcome. *T*-tests were performed to evaluate the association between each dose metric and each outcome. Multiple dose metrics were significantly (p-value < 0.05) associated with early dysphagia, but none were associated with late dysphagia

Four of the five mean dose metrics, pharynx (p = 0.02), PGM_Inf (p = 0.01), PGM_Mid (p = 0.020), and cricopharyngeus (p = 0.01), were significantly associated with early dysphagia, but none were associated with late dysphagia when analyzed using *t*-tests, results are shown in Table 3. The average dose differences in the mean dose for each structure between patients with low-grade (0–2) early dysphagia versus high-grade (3–5) early dysphagia were 7.4 Gy (pharynx), 7.1 Gy (PGM_Mid), 13.0 Gy (PGM_Inf), and 12.3 Gy (cricopharyngeus) respectively. Figure 2 shows the distribution of dose values versus CTCAE grade for each organ.

**Table 3:** T-test results for relationship between dose metrics and early and late dysphagia outcomes

Continuous covariates	T-test p-value	T-test p-value
	(early dysphagia)	(late dysphagia)
Pharynx mean dose	0.02	0.8
PGM_inf mean dose	0.01	0.69
PGM_mid mean dose	0.02	0.68
PGM_sup mean dose	0.09	0.78
Cricopharyngeus mean dose	0.01	0.27

In the multivariate analysis we sought to determine which covariates were the most informative. We achieved this by building multivariate models using a set of covariates (age, sex, smoking history, one dose metric at a time, and either HPV status, cancer type or both). Models were built using both a top-down method (where covariates are removed one by one and re-added if their removal decreases the predictive performance of the model) and using a bottom-up method (where covariates are added one by one and kept if they improve the predictive performance of the model). In our data, both methodologies produced the same model each time except for a model predicting late dysphagia where mean cricopharyngeus dose, HPV status, and cancer type were all included as potential covariates. Results for the p-values are shown in Table 4 while coefficients are in Table 5. For early dysphagia, the models consistently selected cancer type, smoking history, and mean dose as covariates. Of these, mean dose for each structure was significantly associated with outcome and oropharynx status was only significant in models including the mean dose to either the pharynx, PGM_Mid, or PGM_Sup. HPV status, age, and sex were never selected for early dysphagia. For late dysphagia either HPV status or cancer type was selected in each model. The only other covariate that was ever selected for late dysphagia was the mean dose to the cricopharyngeus which was included in the model when oropharynx was not selected. Both HPVstatus and cancer type were significant covariates when they were selected, however no model included both suggesting they contribute similar information to the model.

**Table 4:**
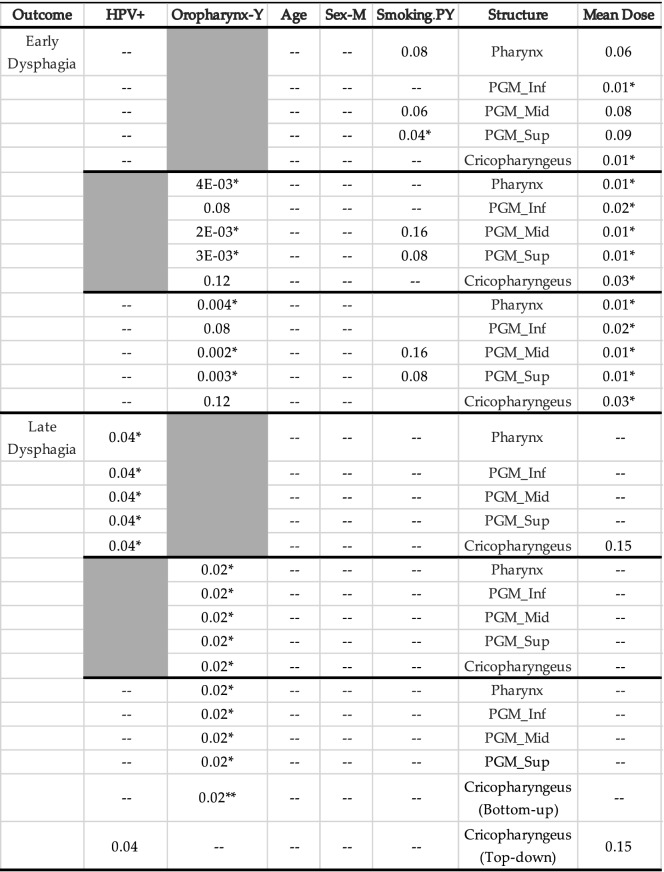
P-values for independent covariates selected in multivariate logistic regression models built using both the top-down and bottom-up methods for early and late dysphagia

Significant (p-value < 0.05) covariates in each model are marked with an asterisk. Greyed out values are those that were not available as potential covariates during model-building

**Table 5:**
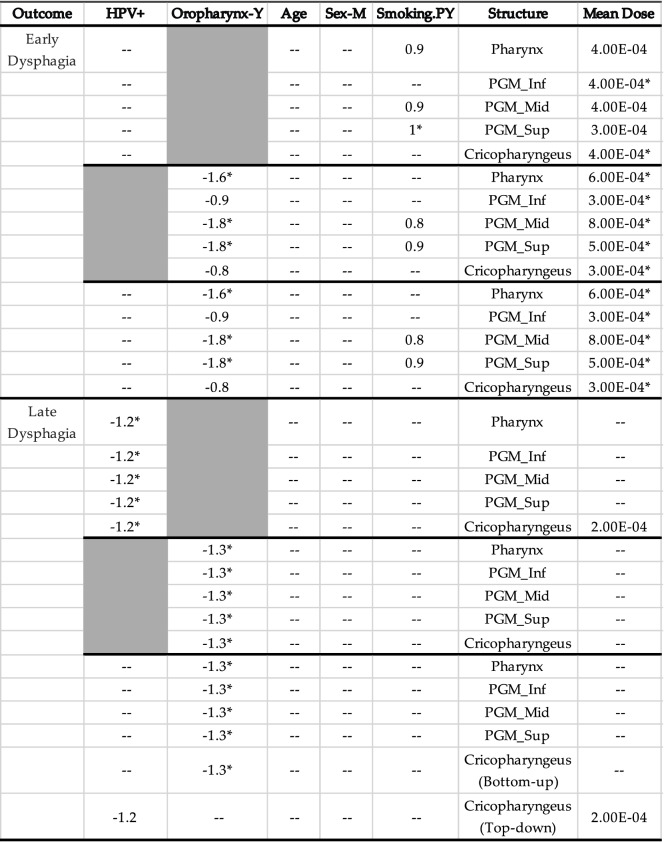
Coefficients for independent covariates selected in multivariate logistic regression models built using both the top-down and bottom-up methods for early and late dysphagia

Significant (p-value < 0.05) covariates in each model are marked with an asterisk. Greyed out values are those that were not available as potential covariates during model-building

## Discussion

In this exploratory study, we sought to determine if HPV status serves as an independent predictor when considered in combination with standard patient characteristics for normal tissue dysphagia outcomes for head and neck cancer patients treated with radiotherapy. We found that for early dysphagia, HPV status was never selected; however, oropharyngeal cancers were associated with worse outcomes as was the mean dose to different structures that are associated with swallowing (e.g., pharynx). Compared to the non-oropharyngeal cancers in this study (e.g., oral cavity, larynx, hypopharynx), the oropharyngeal radiation treatment fields are generally large to treat the primary disease and bilateral nodes. Thus the association between early dysphagia and both dose and cancer type is logical. Additionally we saw that patients with high grade early dysphagia were more likely to have received a dose to the pharyngeal structures greater than 50 Gy which broadly corroborates the threshold recommended in the QUANTEC studies for the entire pharynx. Furthermore, our results suggest that pharyngeal sub-structures have individual thresholds as can be seen in Fig. 2 where there was a significant separation between high and low grade early dysphagia for the inferior pharynx at a threshold of 40 Gy while for the middle and superior pharynx there was significant separation near 60 and 50 Gy respectively. This suggest sub-structure specific DVH goals could be beneficial in reducing toxicity rates. For late dysphagia either HPV status or having an oropharyngeal cancer was consistently selected. These two covariates are known to be heavily associated and thus their alternate selection suggests that they do not provide complementary information to the model but that the use of either one on its own could be informative. Interestingly, for late dysphagia, only the dose to one of the swallowing structures, the cricopharyngeus, was ever associated with worse outcomes. This is a value that is easily evaluated from the initial radiation therapy treatment plan and in a future prospective trial could be monitored on daily adapted plans to see if reducing its value with adaptation decreases the rates of severe toxicity. While HPV is a known contributing factor for tumor prognosis in oropharyngeal cancers, its role in normal tissue toxicities for head and neck cancers has not previously been evaluated although one study showed HPV-positive patients have higher rates of early mucositis [15]. Our results indicate HPV-negative status or having an oropharyngeal cancer may increase a patient’s risk of high-grade late dysphagia. Recent studies have shown that the genomics of the primary tumor can have an effect on normal tissue outcomes [18]. Thus, the relationship seen here between cancer type and worsened outcomes may be due to underlying genetics of the tumor effecting the micro-environment though more analysis is needed to evaluate the cause of this effect.

This study does have notable limitations. Primarily, there may be other covariates that contribute to high-grade toxicities, such as whether patients received surgery or chemotherapy, that were not included in our study. Additionally, our cohort consisted of only 99 patients, and a larger cohort may be needed to establish definitive roles for these covariates in predicting early and late dysphagia.

The goal of this work was to evaluate the role of HPV status on radiation toxicity and to serve as an exploratory platform for evaluating how predictive factors for high grade toxicities can be identified and analyzed in a future prospective study. Identifying patients at high risk from normal tissue toxicities is particularly relevant as we hope to use these criteria to select patients that will most benefit from daily adaptation. Because daily adaptation requires extensive resources from the clinic in terms of machine-hours and presence of experts (physicians and medical physicists) at treatment, it will not be feasible to adapt every patient. As a result, identifying these high-risk characteristics will help us correctly prioritize patients for adaptation. Thus, if HPV status or other patient-specific characteristics can be strongly linked to higher risk of normal tissue toxicity, then patients with those characteristics would be preferentially selected at our clinic for daily adaptation. This would give them the best chance of reducing the dose to those structures while maintaining the high doses to the disease site that are necessary for effective treatment.

## Conclusions

In this study we sought to determine whether HPV status played an independent prognostic role for high grade early and late dysphagia experienced by HNSCC patients. We also evaluated other common patient characteristics and dose metrics from their radiation treatment plans. For early dysphagia, having an oropharyngeal cancer, higher mean doses, and > 10 pack-year smoking history were all associated with higher toxicity. For late dysphagia, the most significant covariate was having an oropharyngeal cancer. We believe these findings highlight the critical importance of additional studies to parse out the individual contributions of primary site and HPV status on the risk of RT-related toxicity with the end goal of facilitating patient prioritization for new technologies such as adaptive RT.

## Data Availability

The datasets used, generated, and analyzed during the current study are available from the corresponding author on reasonable request.

## Abbreviations

CTCAE: Common terminology criteria for adverse events
HNSCC: Head and neck squamous cell carcinoma
HPV: Human papilloma virus
IGRT: Image-guided radiation therapy
IMRT: Intensity modulated radiation therapy
OPSCC: Oropharyngeal squamous cell carcinoma
PGM_inf: Inferior pharyngeal muscles
PGM_mid: Middle pharyngeal muscles
PGM_sup: Superior pharyngeal muscles
RT: Radiotherapy
rtAE: RT adverse event
